# Evaluating the impact of a novel behavioural science informed animation upon breast cancer screening uptake: protocol for a randomised controlled trial

**DOI:** 10.1186/s12889-022-13781-x

**Published:** 2022-07-19

**Authors:** Amish Acharya, Hutan Ashrafian, Deborah Cunningham, Josephine Ruwende, Ara Darzi, Gaby Judah

**Affiliations:** 1 Department of Surgery and Cancer, St Mary’s Hospital, 10th Floor Queen Elizabeth Queen Mother Building, W2 1NY London, UK; 2 Department of Radiology, Imperial College Healthcare NHS Trust, W2 1NY London, UK; 3 NHS England (London Region), London, UK

**Keywords:** Breast cancer screening, Behavioural science, Video messaging, Screening uptake, Healthcare inequalities

## Abstract

**Background:**

Breast cancer screening is estimated to save 1300 lives annually in the United Kingdom. Despite this, uptake of invitations has fallen over the past decade. Behavioural science-informed interventions addressing the determinants of attendance behaviour have shown variable effectiveness. This may be due to the narrow repertoire of techniques trialled, and the difficulties of implementation at a population-scale. The aim of this study is to evaluate the impact on breast screening uptake of a novel behavioural video intervention which can contain more complex combinations of behavioural change techniques.

**Methods:**

A 3-armed randomised controlled trial will be undertaken in London comparing the impact of (1) the usual care SMS reminder, to (2) a behavioural plain text SMS reminder and (3) a novel video sent as a link within the behavioural plain text SMS reminder. A total of 8391 participants (2797 per group) will be allocated to one of the three trial arms using a computer randomisation process, based upon individuals’ healthcare identification numbers. The novel video has been co-designed with a diverse range of women to overcome the barriers faced by underserved communities and the wider population. The behavioural SMS content has also been co-designed through the same process as the video. Messages will be sent through the current reminder system used by the London screening programmes, with reminders 7 days and 2 days prior to a timed appointment. The primary outcome is attendance at breast cancer screening within 3 months of the initial invitation. Secondary outcomes will include evaluating the impact of each message amongst socio-demographic groups and according to the appointment type e.g. first invitation or recall.

**Discussion:**

In addition to general declining trends in attendance, there is also concern of increasing healthcare inequalities with breast cancer screening in London. The current novel intervention, designed with underserved groups and the general population, incorporates several behavioural techniques to overcome the barriers to attendance. Understanding its potential impact in a real-world setting therefore may provide significant information on how to address reducing attendance and healthcare disparities.

**Trial Registration:**

This study was registered on ClinicalTrials.gov (NCT05395871) on the 27^th^ May 2022.

**Supplementary Information:**

The online version contains supplementary material available at 10.1186/s12889-022-13781-x.

## Background

The National Health Service Breast Screening Programme (NHSBSP) invites women aged 50 to 70 for a mammogram every three years. By enabling earlier detection of breast cancer, it is estimated to save 1300 lives per year [1]. Despite this, uptake of invitations has fallen, with rates in 2019/20, prior to the pandemic, below the acceptable threshold, with 69.1% coverage [2]. COVID-19 has exacerbated these trends. Almost 1 million mammograms were missed, and there was a 39.2% decrease in the numbers of women who had cancers detected through screening in 2020/21 compared the previous year [3]. Moreover, significant healthcare inequalities have been reported with breast cancer screening, with those from minority ethnic groups, more deprived areas and suffering multiple medical conditions less likely to attend [4–7]. Addressing these challenges, has therefore, become a significant public health concern.

Behavioural science is a field that investigates and addresses the socio-psychological constructs that explain health behaviours. Several interventions informed by behavioural science have already been trialled to facilitate breast screening attendance [8, 9]. Whilst some of these have been successful, such as Short Message Service (SMS) reminders leading to an increase in uptake by 5%, others have not improved attendance [10]. A recent systematic review into breast cancer screening interventions reported only 50% were effective [11]. There are several reasons for this low level of success. For example, some interventions are designed to target behavioural determinants exhibited in subgroups which are not as prevalent amongst the general population, and thus assume subgroup homogeneity [12]. In addition, there are also often issues implementing novel interventions feasibly into real-world population-level programmes. These factors need consideration when developing novel interventions to improve breast screening uptake.

Currently, invitation letters and SMS reminders are the predominant means of communication between the NHSBSP and women due for screening [13]. Recent United Kingdom (UK) guidance on how mobile messaging can be effectively incorporated into population screening programmes has provided recommendations on how to optimise this means of communication to facilitate attendance [14]. This includes the use of behavioural science-informed messages. However, this guidance also acknowledges that plain text-based messages often need to conform to restrictions on the length and the content delivered. Videos, however, can enable more complex behavioural content to be employed without convoluting wording [15]. Moreover, they can enable a broader range, and unique combinations of Behavioural Change Techniques (BCTs) to be employed [16], so that a greater breadth of determinants amongst a wider population can be addressed. There is, however, a paucity of research to investigate how effective such messaging is at improving uptake in a population-based breast cancer screening programme.

This protocol describes an exploratory 3-armed randomised controlled trial (RCT) which aims to investigate the impact of an SMS reminder with a link to a novel behavioural science-informed video upon the uptake of breast cancer screening invitations, compared to a text-only behavioural science informed SMS message and the usual care SMS reminder.

## Methods

### Study design

The study is a 3-armed RCT comparing the effect of (1) a behavioural SMS reminder with a link to a novel behavioural science informed video (2) the behavioural SMS reminder without the behavioural video and (3) the usual care reminder SMS. As a result of the COVID-19 pandemic in which screening was ceased, services across England have had to deal with a backlog of missed mammograms. To prevent services becoming overwhelmed, and to aid recovery, breast cancer screening began utilising open invitations [17]. These messages invited eligible women to contact the screening service to book an appointment, as opposed to the traditional method of offering a mammogram at a pre-determined time, which could be rearranged. As the NHSBSP continues to recover in the wake of the pandemic, services in London have adopted a hybrid approach involving both traditional timed and open invitations, using two types of usual care reminders depending on the invitation type. As a result, the decision was made to develop both the video and behavioural science SMS message content so that they are relevant to and adaptable to either timed or open invitations while encompassing the same BCTs (Additional file 1).

**Fig. 1:**
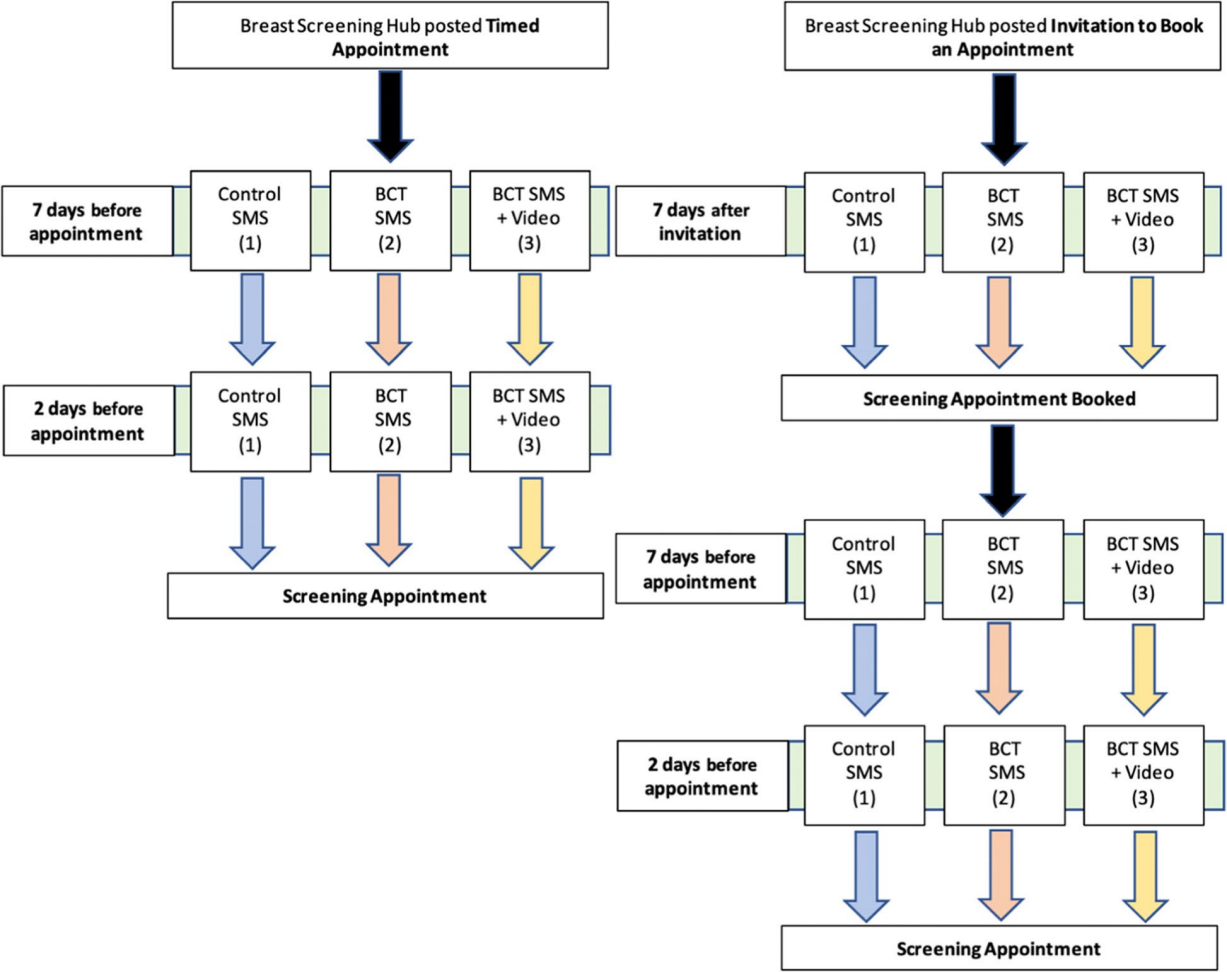
Figure demonstrating the messaging schedule and trial arms included within the randomised control trial

The trial will be conducted with the NHS Breast Screening Programme in London. This region composes of 6 screening services but is administered from a singular hub [18]. The region traditionally has a low attendance at breast screening, and currently has the lowest uptake of invitations in the England [3]. It is also an area with a highly diverse socio-demographic population. This clinical trial has been prospectively registered on ClinicalTrials.gov (identifier NCT05395871) and has been granted a favourable ethical opinion from London- Surrey Research Ethics Committee (reference 22/LO/0325). The study design is detailed in Fig. 1.

### Participants

Participants will be those invited to attend the NHSBSP in participating London services during the study period. Inclusion criteria parallel the eligibility criteria of the screening programme with women aged 50 to 70, who have not had a mammogram in the previous 3 years, and have not a double mastectomy, enrolled [19]. Women will not be actively consented to participate in this study, as the presence of an explicit consent process may increase salience of the programme and thus affect health behaviours. In keeping with previous behavioural studies in screening an implied consent model will be used, with those who have not opted out of receiving messaging randomised [20].

Based upon previous studies, and what would constitute a clinically meaningful effect size a power calculation has been conducted. Assuming 5% type 1 error probability, 80% power, and an effect size of 3% increase in either intervention arm compared to the control, a minimum sample size of 2797 people per study arm (8391 in total) will be recruited. As an estimated 30 to 40,000 screening invitations are sent per month across all 6 London screening services, the approximated sample size is a feasible recruitment target for the current study over an estimated 2-month recruitment period.

Eligible women will be randomised to either one of the 3 trial arms using a computer-based system. The computer algorithm will allocate participants based upon the last two digits of the individual’s NHS number, which is a unique identifier allocated to an individual on registration with the UK’s health service. The allocation ratio will be 33:33:34, video intervention: SMS intervention: control. All participants invited to screen have an NHS number. This method enables randomisation at the level of the individual and ensures that women who reschedule appointments remain within the same trial arm. Allocation will be undertaken by the direct care team using current screening infrastructure, with researchers blinded to this allocation.

### Interventions

Currently the NHSBSP sends out a letter invitation followed by an SMS reminder at 7 days, and a further message 48 h prior to a booked appointment. In addition, for an open invitation, a reminder to book is sent 7 days following the initial letter. For each arm of the trial the schedule will be kept the same and will be sent according to the usual protocol for times or open invitations. These usual care message reminders, provide information regarding the upcoming appointment such as location, time and date. Furthermore, they provide a ‘usual care’ link to further online information which includes a video providing details of the programme but does not contain specific BCTs to facilitate attendance.

The intervention video will be sent via SMS message using a new web address to replace the usual care link. This short video animation has been developed using an evidence-based and co-design process, to overcome the common barriers identified amongst under-served groups including minority ethnic groups, from areas of higher deprivation, those with mental health illness and multiple medical problems. The creation of this novel innovation is described in full elsewhere [21]. In summary, it was developed following 10 interviews and 2 focus groups which purposively recruited women from under-served populations. Following an inductive thematic analysis of transcripts, the barriers and facilitators to attendance, such as fear of a cancer diagnosis or lack of perceived susceptibility, were mapped to the Theoretical Domains Framework (TDF) [22] and Capability Opportunity Motivation- Behaviour Model (COM-B) [23]. These findings were then triangulated with the results from a systematic review, and a previous survey of 1000 women in London exploring psychological determinants of breast cancer screening non-attendance [24]. The themes from each of these sources were also mapped onto the TDF and COM-B. The triangulation exercise elicited the common barriers and facilitators to breast screening. By including barriers amongst under-served groups as well as those highlighted by the wider population, subgroup homogeneity was not assumed, and a wide range of barriers and facilitators reported by women from a range of backgrounds were considered. Potential BCTs to overcome the common barriers to screen were then derived using the Theory and Techniques Tool [25]. This candidate list of BCTs was then used as the basis for activities in four co-design workshops. A purposive sampling technique was used to recruit to these workshops to ensure representation from a wide range of service users. Workshops used different types of activities to (a) validate the findings of the triangulation exercise, (b) highlight which candidate BCTs to use within the video and behavioural SMS, and (c) develop imagery and wording that best expressed these BCTs for the interventions and combining these to develop an initial storyboard. Through an iterative process of feedback including extensive patient and public involvement, which involved consultation with organisations such as the Oremi Centre and Asian Woman Cancer Group, and with behavioural scientists with expertise in screening, the storyboard was refined. Additional stakeholders including NHS commissioners, screening services, clinicians specialising in breast cancer care, NHS identity and the communications teams from two London NHS trusts, also gave feedback through this development process. The finalised animation, which was approved by stakeholders, was then translated into thirteen languages, with new voiceovers provided in an additional three languages to ensure it could be understood by a diverse population.

The novel behavioural science-informed video will be sent via a weblink replacing the usual care link in SMS reminders. The content of the SMS containing the new video link will be the same as that within the plain-text behavioural SMS. Both video and behavioural SMS therefore will contain the same BCT content within the SMS itself, which has been developed through the evidence synthesis and feedback activities described previously. The behavioural SMS will contain the usual care link.

### Outcomes

The primary outcome measure will be attendance at breast cancer screening appointment, as reported by the screening record, 3 months following the invitation letter. This timeframe is in keeping with similar studies examining the effect of behavioural interventions in cancer screening [26, 27]. Secondary outcomes will examine the impact of interventions upon uptake amongst subgroups (Table 1). Furthermore, an online questionnaire will be used to ascertain the perspectives of women who receive the behavioural video, regarding its influence upon their intention to attend screening (Additional file 2). It should be noted that no power calculations have been made for these secondary measures. Booking and attendance measures will also be collected as part of an interim analysis (T_i_) undertaken half-way through the study. This will be used to ensure uptake is not being significantly negatively impacted and below expected levels leading to cessation of the trial. This data will be shared with screening service leads and the hub, who are independent from researchers and will form part of the monitoring committee, as part of their usual role in assessing uptake rates for breast services in London. Any adverse events or harms will be reported to the screening hub, as well as Imperial College London as the primary sponsor. Data collection will be undertaken through the screening services NHS Breast Screening System, which collects demographic variables, as well as information on attendance. Data of all recruited participants will be extracted by a member of the direct care team from this system, and pseudo-anonymised (removing identifiers) to send securely to the encrypted data storage facility at Imperial College London. In this way, analysis can be undertaken with researchers being blinded to the allocation of individuals, and confidentiality maintained as personal information is not being sent outside of the direct care team.

**Table 1:** Demonstrating the outcome measures and the differing timepoints at which these are expected to be collected

Outcome Measure	Definition	Timepoint Collected (T_0_, T_i_, T_e_)
Age	Age at the time of the initial invitations	T_o_
Ethnicity	Ethnicity code according to record based upon 2011 Census	T_o_
IMD	Level of deprivation based upon postcode	T_o_
Appointment Type	Open or timed invitation	T_o_
SMS allocation	Usual Care, Behavioural SMS or Behavioural SMS with new video	T_o_
Location of invitation	Location of screening service in which appointment is due within London	T_o_
Invitation designation	First invitation or routine recall appointment	T_o_
Booked appointment	Whether an open invitation has been booked	T_i_, T_e_
Attendance at appointment	Whether an individual has attended the appointment	T_i_, T_e_
Message Sent	Whether a message was sent or was unable to be sent (e.g. wrong number)	T_e_
Perspectives upon novel video	Collected via an online questionnaire available to participants allocated to the Behavioural SMS + video arm (questionnaire accessed on same webpage as video)	T_e_

*T*_*0*_ Baseline, *T*_*i*_ Time of Interim Analysis, *T*_*e*_ Time of Endpoint Analysis

### Analysis

Data will be reported in accordance with Consolidated Standards of Reporting Trials (CONSORT) guidelines. Data will be extracted from the screening service database by a third party, and identifying details will be removed before being sent securely to the researchers. Data analyses will be undertaken by researchers blinded to the allocation throughout the study. This will be achieved through removing NHS identifiers and allocation code prior to transfer of data by an independent member of the screening team. A planned interim analysis will be undertaken with regard the primary outcome midway through the trial. The aim of this analysis it to ensure that one of the messages does not unexpectedly lead to a significant reduction in uptake. Whilst this is unlikely given the extensive feedback processes used to develop the messages, this will ensure ongoing acceptability. The percentage uptake of screening invitations by women who received differing messages will be compared. Both intention-to-treat (ITT) and per protocol (PP) analysis will be undertaken, with the latter referring including only those women who received the message. This will be ascertained through message delivery receipts. For the main analysis at the end of the study, we will utilise hierarchical regression modelling to determine the impact of each trial arm upon uptake, adjusting for the type of invitation (open versus timed) individuals were sent and socio-demographic groups (e.g. high versus low deprivation and older versus younger). These covariates will only be retained within the model if their inclusion leads to a significant improvement in the fit.

Secondary outcomes will also investigate attitudes towards the novel behavioural video from the online questionnaire (Additional file 2), including whether the video impacted upon intention to attend. Quantitative response will be aggregated into frequency distributions. Free-text responses will be transcribed and coded by two independent authors using a constructivist approach to derive themes. This inductive thematic analysis will be conducted to understand the key themes regarding the public’s perception of the novel video and its influence upon attendance.

## Discussion

The NHS Long-Term Plan has set a target to diagnose 75% of cancers at stage 1 or 2 by 2028 [28]. Screening is key to achieving these aims by enabling earlier detection of cancer, at an asymptomatic stage. The success of any screening programme, however, relies upon sufficient uptake of invitations to screen. The uptake of breast cancer screening has been falling. In addition, the programme faces several challenges including a backlog of invitations due to disruptions from Covid-19, and growing healthcare inequalities [14, 29].

This study, to our knowledge, is the first to examine the impact of a novel video intervention, developed using behavioural science approaches, integrated into the NHS Breast Cancer Screening Programme’s invitation structure. The use of video-based interventions in screening has been poorly studied. Their use may facilitate the incorporation of multiple behavioural techniques, and therefore potentially have a broader effect than plain-text messages. Including a link to a video within an SMS does not incur any additional costs to the screening service, and if successful, can be quickly and easily rolled out. Moreover, the versatility of the medium can be translated into several different areas such as social media, which has been shown to impact upon health behaviours including screening attendance [30]. This study will look to provide real-world evidence regarding the effectiveness of the behavioural SMS and video interventions, and potentially will have significant public health applications regarding ways to facilitate cancer screening uptake.

## Supplementary Information

**Additional file 1.** Table demonstrating the content of SMS messages sent in different arm, and by invitation type (open v. timed). Wording highlighted in yellow represents included behavioural change techniques. Wording highlighted in green represents the new video link.

**Additional file 2.** Video Feedback Questionnaire

## Data Availability

Data sharing is not applicable to this article as no datasets were generated or analysed during the current study at this stage. Data from the trial will be available from authors upon reasonable request.

## Abbreviations

BCT: Behavioural change technique
COM-B: Capability, opportunity, motivation- behaviour model
CONSORT: Consolidated standards of reporting trials
COVID-19: Coronavirus disease 2019
IMD: Index of multiple deprivation
ITT: Intention to treat
NHS: National health service
NHSBSP: National health service breast screening programme
*PP*: Per protocol
RCT: Randomised controlled trial
SMS: Short message service
TDF: Theoretical domains framework
UK: United kingdom
