# Supplementary material for: Evaluating the impact of a novel behavioural science informed animation upon breast cancer screening uptake: protocol for a randomised controlled trial
Source: BMC Public Health. 2022 Jul 19;22:1388. doi: 10.1186/s12889-022-13781-x (PMC9295097; doi:10.1186/s12889-022-13781-x)
Supplement: Supplementary file 1 — Additional file 1. Table demonstrating the content of SMS messages sent in different arm, and by invitation type (open v. timed). Wording highlighted in yellow represents included behavioural change techniques. Wording highlighted in green represents the new video link. [file 12889_2022_13781_MOESM1_ESM.docx]

| **Reminders at 7 and 2 days prior to appointment** | | |
| --- | --- | --- |
| **a) Control/Usual Care** | **b) Behavioural SMS** | **c) Behavioural SMS + Video** |
| *Don't forget your breast screening appointment is at 10:36 am on 26/10/2021 at Edgware Community Hospital.*  *To re-arrange or cancel your appointment call ___________or visit www.london-breastscreening.org.uk*  *For further info please click here: https://_____________* | *Following the breast cancer screening letter you received, don’t forget your appointment is at 10:36am on 26/10/2021 at Edgware Community Hospital.*  *Detecting breast cancer early gives you the best chance to fully recover.*  *To find out why we’re inviting you and what to expect watch this video: https://___________*  *To re-arrange or cancel your appointment call ___________* | *Following the breast cancer screening letter you received, don’t forget your appointment is at 10:36am on 26/10/2021 at Edgware Community Hospital.*  *Detecting breast cancer early gives you the best chance to fully recover.*  *To find out why we’re inviting you and what to expect watch this video: https://__________*  *To re-arrange or cancel your appointment call ___________* |

**Message Content**

Table demonstrating the content of SMS messages sent in different arm, and by invitation type (open v. timed). Wording highlighted in yellow represents included behavioural change techniques. Wording highlighted in green represents the new video link.

| **Message sent 7 days after the open invitation to book an appointment** | | |
| --- | --- | --- |
| **a) Control/Usual Care** | **b) Behavioural SMS** | **c) Behavioural SMS + Video** |
| *Breast Screening appointments have changed. You will have received a letter on how to book an appointment. Please call the London Breast Screening Hub___________ or visit* [*www.london-breastscreening.org.uk*](http://www.london-breastscreening.org.uk) *to arrange an appointment at your nearest location. For further info please click here: https://_____________* | *Breast Screening appointments have changed.  You will have received a letter on how to book an appointment. Please call the London Breast Screening Hub on ___________or visit www.london-breastscreening.org.uk to arrange an appointment locally.*  *Detecting breast cancer early gives you the best chance to fully recover. To find out why we’re inviting you and what to expect watch this video: https://_____________* | *Breast Screening appointments have changed.  You will have received a letter on how to book an appointment. Please call the London Breast Screening Hub on ___________ or visit www.london-breastscreening.org.uk to arrange an appointment locally.*  *Detecting breast cancer early gives you the best chance to fully recover. To find out why we’re inviting you and what to expect watch this video: https://__________* |
