# Supplementary material for: Evaluating the impact of a novel behavioural science informed animation upon breast cancer screening uptake: protocol for a randomised controlled trial
Source: BMC Public Health. 2022 Jul 19;22:1388. doi: 10.1186/s12889-022-13781-x (PMC9295097; doi:10.1186/s12889-022-13781-x)
Supplement: Supplementary file 2 — Additional file 2. Video Feedback Questionnaire [file 12889_2022_13781_MOESM2_ESM.docx]

Additional File 2

Video Feedback Questionnaire

Q2 How old are you?

- 50 to 55
- 56 to 60
- 61 to 65
- 66 to 70
- Prefer not to say

Q3 Which best describes your ethnic background?

- Arab
- Asian or Asian British
- Black, African, Caribbean, or Black British
- Mixed or Multiple Ethnicity
- White
- Any other ethnic group
- Prefer not to say

Display This Question:

If Q3 = Any other ethnic group

Q4 If any other ethnic group, please specify

________________________________________________________________

Q5 Have you been invited to attended breast screening in the past?

- Yes
- No
- Prefer not to say

Display This Question:

If Q5 = Yes

Q6 How often have you attended breast cancer screening in the past?

- Never attended
- Sometimes attended
- Always attended

|  | 0 | 1 | 2 | 3 | 4 | 5 | 6 | 7 | 8 | 9 | 10 |
| --- | --- | --- | --- | --- | --- | --- | --- | --- | --- | --- | --- |
| How much did you learn about breast cancer/breast cancer screening from watching the video? | No information was new |  |  |  |  | Some of the information was new |  |  |  |  | All the information was new |
| How relatable did you feel the breast cancer screening stories were? | Extremely Unrelatable |  |  |  |  | Neither relatable nor not |  |  |  |  | Extremely relatable |
| What were your thoughts regarding attending breast cancer screening BEFORE watching the video? | Not planning to go |  |  |  |  | Undecided |  |  |  |  | Definitely planning to go |
| Has the video changed your opinion on attending breast cancer screening? | Made me a lot less likely to go |  |  |  |  | No change |  |  |  |  | Made me a lot more likely to go |
| How likely are you to recommend the video to a friend, family member or colleague? | Not at all likely |  |  |  |  | Neutral |  |  |  |  | Extremely likely |

Q10 Which story did you feel was the most influential on you?


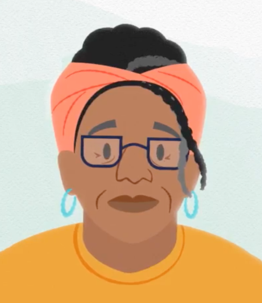


Faith


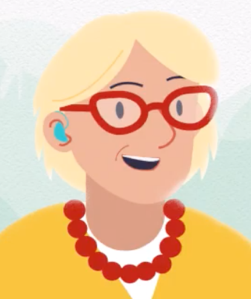


Martha


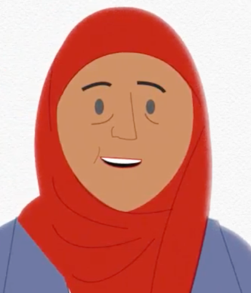


Aleema


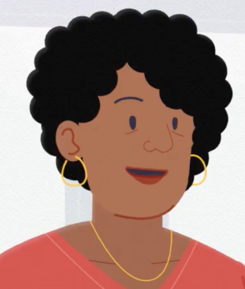


Devi


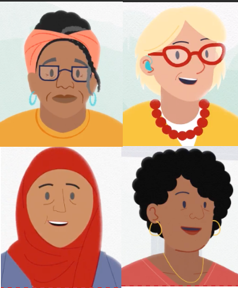


All of them

Q13 If you have any further comments about the video, including suggested improvements, please write them below.

________________________________________________________________

________________________________________________________________

________________________________________________________________

________________________________________________________________

________________________________________________________________
